# Recent advances in understanding the regulation of metalloproteinases

**DOI:** 10.12688/f1000research.17471.1

**Published:** 2019-02-18

**Authors:** David A. Young, Matt J. Barter, David J. Wilkinson

**Affiliations:** 1 Skeletal Research Group, Institute of Genetic Medicine, Central Parkway, Newcastle University, Newcastle upon Tyne, NE1 3BZ, UK

**Keywords:** metalloproteinase, transcription, cartilage, osteoarthritis, regulation

## Abstract

Metalloproteinases remain important players in arthritic disease, in part because members of this large enzymatic family, namely matrix metalloproteinase-1 (MMP-1) and MMP-13, are responsible for the irreversible degradation of articular cartilage collagen. Although direct inhibition of MMPs fell out of vogue with the initial clinical disappointment of the first generation of compounds, interest in other mechanisms that control these important enzymes has always been maintained. Since these enzymes are critically important for tissue homeostasis, their expression and activity are tightly regulated at many levels, not just by direct inhibition by their endogenous inhibitors the tissue inhibitors of metalloproteinases (TIMPs). Focussing on MMP-13, we discuss recent work that highlights new discoveries in the transcriptional regulation of this enzyme, from defined promoter functional analysis to how more global technologies can provide insight into the enzyme’s regulation, especially by epigenetic mechanisms, including non-coding RNAs. In terms of protein regulation, we highlight recent findings into enzymatic cascades involved in MMP-13 regulation and activation. Importantly, we highlight a series of recent studies that describe how MMP-13 activity, and in fact that of other metalloproteinases, is in part controlled by receptor-mediated endocytosis. Together, these new discoveries provide a plethora of novel regulatory mechanisms, besides direct inhibition, which with renewed vigour could provide further therapeutic opportunities for regulating the activity of this class of important enzymes.

## Introduction

Osteoarthritis (OA), affecting millions of people worldwide, is the most prevalent arthritic disease. The aetiology of the disease is complex and it has a number of risk factors but age predominates; in fact, in the UK, more than one in three adults over 45 years old have sought treatment for OA ^1^. OA occurs in an insidious manner with distinct molecular pathways resulting in progressive cartilage loss, osteophyte formation, subchondral bone thickening and often a degree of synovial inflammation. There are currently no effective treatments which alter disease progression.

Although OA is a disease of the whole articulating joint, the proteolytic destruction of cartilage, especially of type II collagen, is a central and irreversible process that underpins OA. Cartilage is composed predominantly of type II collagen and the proteoglycan aggrecan. Collagen loss is particularly important because of its slow turnover, making this process essentially irreversible ^2^. Collagens are defined by their unique triple-helical structure which limits their susceptibility to cleavage at a single site in the triple helix (PQG _775_↓ _776_LAG) to a very specific group of proteinases ^3^. With antibodies raised against the C-terminal neoepitopes generated by proteinase cleavage at this region, an increase in immuno-staining has been observed in aged cartilage and even more so in OA cartilage ^4– 6^. Along with this wealth of human data, recent strong evidence that this specific cleavage is essential in cartilage destruction comes from transgenic mice with mutated amino acids around the cleavage site in collagen (to PPG _775_↓ _775_-MPG), which blocks proteolysis at the primary collagenase cleavage site. This mutation had no impact on normal collagen fibrillogenesis, but heterozygous collagen cleavage-resistant mice, when subjected to the surgically induced post-traumatic OA model, destabilisation of the medial meniscus (DMM), were highly protected ^7^. In terms of the proteinases involved, it is well established that it is the matrix metalloproteinases (MMPs), especially the soluble collagenases MMP-1, MMP-8 and MMP-13, that are crucial for this destruction to occur, and prevailing dogma suggests that, in OA, MMP-13 predominates ^3^. Other MMPs such as MMP-2 and MMP-14 have reported collagenolytic activity but their contribution to cartilage pathology is likely minor. MMP-3, MMP-9 and MMP-10 degrade other extracellular matrix (ECM) components but, *in vivo*, are unable to cleave native type II collagen. Again, the importance of MMP-13 in type II collagen cleavage is supported by the DMM-OA model when performed in *Mmp13 ^−/−^* mice. In this model, *Mmp13 ^−/−^* mice show less tibial cartilage erosion than do wild-type mice at 8 weeks post-surgery ^8^. Conversely, cartilage-restricted expression of a constitutively active MMP-13 in mice induces a joint pathology that strongly resembles OA ^9^.

Mice deficient in MMP-13 are grossly indistinguishable from wild-type animals and have normal fecundity, and a normal lifespan and no overt phenotypic abnormalities ^10, 11^. However, when challenged, the mice do show increased collagen deposition in the intima of aortic lesions ^12^, whereas upon full-thickness cutaneous wounding, *Mmp13 ^−/−^* mice have delayed re-epithelialisation ^13^. Together, these studies show that MMP-13 has a role in atherosclerosis and would healing, highlighting a role for the enzyme beyond cartilage. In terms of skeletal development, histological analysis of developing *Mmp13 ^−/−^* animals shows an expanded growth plate, which is due to enlargement of the hypertrophic zone. The animals thus show a profound delay in development of the primary ossification centre, which begins to normalise after birth ^10, 11^. Interestingly, in skeletally mature animals, both the tibial and femoral growth plates of *Mmp13 ^−/−^* mice have focal regions of bony union, something unseen in wild-type littermates ^8^. Many of the growth plate features of the *Mmp13 ^−/−^* mice are consistent with the human chondrodysplasia group metaphyseal anadysplasia 1 (which includes Missouri-type spondyloepimetaphyseal dysplasia) and are caused by a mutation in MMP-13 and can improve spontaneously by early adolescence ^14– 16^.

Together, these observations highlight MMPs, and especially MMP-13, as critical players in cartilage collagen destruction. Moreover, MMP-13 can cleave a wealth of other matrix molecules, including type IV and IX collagen, perlecan, osteonectin and proteoglycans ^17^. Given this, a large body of work and studies revolved around generating and testing chemical inhibitors of MMPs. However, selective targeting of MMPs, including the collagenases, represents a significant challenge as they exhibit a high degree of structural similarity across their active sites ^3, 18^. Indeed, owing to poor selectivity, many MMP inhibitors displayed off-target effects in clinical trials and had noticeable side effects, including joint arthralgia ^19^. Thus, for a long period, MMP-13 inhibition has been out of vogue. However, recent developments are beginning to allow the prospect of selectively removing MMP-13 activity from OA cartilage, be it biologically, biochemically or genetically. In the following sections, we discuss recent publications characterising mechanisms of MMP regulation at these various levels and focus on MMP-13.

## Regulation by *MMP13* transcription

Evolution of *MMP* family members occurred via gene duplication predisposing commonalities in promoter sequence and regulation ^20^. Many MMPs, especially those duplicated in the human chromosomal region 11q22, have well-defined promoter elements with a conserved TATA sequence at about -30 base pairs (bp) and an AP-1 binding site at about -70 bp ^21, 22^. *MMP1* and *MMP13* also possess an ETS-domain transcription factor PEA3‐binding site adjacent to the proximal AP-1 site ^21, 22^. Additional AP-1 sites are present in many MMP promoters.

For many years, it has been shown that numerous stimuli induce the expression of *MMPs* in cartilage, including pro-inflammatory cytokines such as interleukin-1 (IL-1), IL-6, IL-17, and tumour necrosis factor alpha (TNFα) as well as pleiotropic cytokines such as oncostatin M (OSM) and growth factors ^23– 27^. Many of these cytokines and growth factors trigger intracellular signalling pathways, such as the extracellular signal–regulated kinase (ERK), Jun N-terminal kinase (JNK) and p38 mitogen-activated protein kinase (MAPK) pathways, causing expression and activation of AP-1 factors c-Jun and c-Fos and ETS transcription factor family members to directly induce *MMP* transcription ^28, 29^. Nuclear factor-kappa B (NF-κB) pathway activation of IκB releases p50(NF-κB1)/p65(RelA) to activate *MMP* gene expression ^28^. However, even given this long-standing knowledge, the mechanism by which these cytokines and transcription factors directly impact on MMP expression has proven to be somewhat elusive.

Recently, the binding of c-Fos to the *MMP13* proximal promoter in articular chondrocytes was confirmed via chromatin immunoprecipitation (ChIP), but only transiently at an early time point (1 hour), inconsistent with the later *MMP13* induction (6–24 hours) ^30^. Instead, c-Fos was proposed to mediate induction of ATF3, which itself goes on to bind to the *MMP13* proximal AP-1 site to regulate the gene ^30^. In a subsequent study, Baker *et al*. describe how IL-1, combined with OSM, acts via protein kinase D3 (PKD3), downstream of protein kinase C (PKC) signalling, to regulate ATF3 expression and therefore *MMP1* and *MMP13* expression in chondrocytes ^31^.

Toll-like receptor (TLR) agonists such as RNA/DNA and matrix components or fibronectin fragments also induce MMP expression through activation of MAPK and NF-κB pathways ^32– 34^. Previously, protein kinase R (PKR), which is activated by dsRNA, had been implicated in cytokine-mediated gene expression in chondrocytes ^35^. Ma *et al*. now show that TNFα-induced phosphorylation of PKC is dependent on PKR which subsequently activates NADPH oxidase generation of reactive oxygen species and the ERK and NF-κB signalling pathways ^36^. This ERK pathway activation also prevented the peroxisome proliferator-activated receptor gamma (PPARγ)-mediated inhibition of *MMP13*, which has previously been described ^37^. It remains unclear how TNFα activates PKR in chondrocytes, but interactions between TNF receptor-associated factor (TRAF) and PKR are known ^38^. Separately, Ma *et al*. showed that advanced glycation products cause suppression of PPARγ levels to upregulate *MMP13* expression ^36^.

The IL-1 induction of *MMP13* expression is also partially dependent on zinc transporter Zip8-mediated zinc influx ^39^. Increased zinc induces *MMP13* expression, and expression of other MMPs, mediated to some extent by Mtf1 although other transcription factors are also activated ^39^. Mtf1 overexpression is able to induce *MMP13* expression, but no direct interaction with the *MMP13* promoter was explored ^39^. Stress-inducible nuclear protein 1 (Nupr1) was also recently implicated in the IL-1 induction of MMP-13 expression ^40^. Elsewhere, Nupr1 was identified in combination with c-Jun at the *MMP13* promoter ^41^.

During endochondral ossification, MMP-13 expression is tightly restricted to chondrocytes of the lower zone of hypertrophic cartilage which also express type X collagen ^42^. Examining what processes contribute to the hypertrophy of chondrocytes characteristic of OA, Bianchi *et al*. demonstrate that FGF23, which is upregulated in human OA cartilage, induces expression of *MMP13* in chondrocytes via FGF receptor 1 ^43^. α-Klotho acts as a co-receptor for FGF23 but was not required in this context; however, Chuchana *et al*. determined that treatment of human chondrocytes with recombinant α-Klotho or α-Klotho–targeting small interfering RNA (siRNA) suppressed or induced *MMP13* expression, respectively ^44^. The mechanism was not examined, but elsewhere α-Klotho is reported to disrupt signalling pathways, including Wnt, transforming growth factor-beta (TGF-β) and insulin-like growth factor (IGF) ^44, 45^. The authors suggest a chondroprotective role for α-Klotho in OA, although more detailed *in vivo* experiments are needed ^44^. Notch signalling plays a key role in skeletal development where it can regulate chondrocyte differentiation ^46^. Notch may also play a role in OA development, and Sugita *et al*. showed that Hes1, a transcription factor and important target of Notch signalling, can directly induce *MMP13* expression via binding to a regulatory element within intron 4 of the gene ^47^.

Genomic enhancer regions can function across large distances and are emerging as key elements for transcriptional regulation in skeletal biology and development ^48^. Gene expression is directed by the interplay of different enhancers and tissue-specific promoter elements, such as the osteoblast-specific element upstream of *MMP13* transcription start site ^21^. Previously, AP-1 ChIP-sequencing (ChIP-seq) identified a -20 κB IL-1–responsive enhancer regulating *MMP13* expression in chondrocytes ^49^. More recently, Meyer *et al*. used ChIP-seq to identify -10 κB and -30 κB upstream transcription factor binding enhancer sites required for vitamin D receptor and basal *MMP13* expression, respectively, in osteoblasts ^50, 51^. Large consortia projects such as ENCODE and Roadmap have characterised the genomic landscape to identify the whereabouts of histone modifications and transcription factors in numerous cell types ^52^. Until recently, the chondrocyte landscape remained unexplored, but Liu *et al*. now present ATAC-seq (assay for transposase-accessible chromatin using sequencing) mapping of accessible chromatin regions in chondrocytes ^53^. From the reported data, transcription factor binding sites inferred by accessibility for *MMP1* and *MMP13* include C/EBP:AP-1, STAT3/4, NF1 and Fra1 ^53^. These intriguing findings now require experimental validation.

Countering the induction of *MMPs*, anabolic growth factors such as TGF-β, IGF1, interferon gamma (IFNγ) and retinoic acid can repress the expression of *MMP13*, often by repression of intermediate transcription factor induction or activation or by direct competition for binding sites with AP-1 ^28, 54, 55^. Santoro *et al*. recently showed that treatment of cells with recombinant serine proteinase inhibitor SERPINE2 (protease nexin-1) also inhibits IL-1–induced expression of MMP-13 as well as ERK and NF-κB pathway activation and c-Jun levels, although the mechanism of the SERPINE2 effect was not explored ^56^.

Mechanical loading of cartilage in the form of compression, tension and shear also regulates *MMP* expression ^57^. Overloading causes upregulation of MMPs and cartilage degradation, whereas moderate physiological levels of loading on cartilage are considered critical for maintaining cartilage integrity ^58^. In fact, moderate compression appears to repress *MMP1* and *MMP13* expression by upregulating mechanosensitive transcriptional co-regulatory CITED2 ^59^, the activation of which occurs via the primary cilia and subsequent ERK MAPK pathway activation ^60^. However, the beneficial effect of physiological loading on cartilage may be mediated more by upregulating anabolic activity, as an assessment of the literature by Bleuel *et al*. implies that there is little downregulation of proteases under any loading conditions ^57^.

The role of epigenetic mechanisms, including histone modifications and DNA methylation, in *MMP* expression is well documented ^61^. Histone deacetylase (HDAC) inhibitors regulate both basal and cytokine-induced *MMP* expression, in general repressing the cytokine-induced expression of *MMP1* and *MMP13* ^62, 63^. Previously, the class III HDAC SIRT1 has been demonstrated to promote cartilage anabolism and enhance chondrocyte survival *in vitro* and *in vivo*. Elayyan *et al*. extended their previous studies by examining the role of SIRT1 in catabolic chondrocyte gene expression ^64^. IL-1 activated Wnt signalling and induced LEF1 levels to upregulate *MMP13*, processes that are counteracted by SIRT1 which directly represses LEF1 expression, although the mechanism remains elusive ^64^. GDF5 plays a key role in joint development, and the GDF5 locus harbours polymorphisms associated with OA but its function in cartilage homeostasis is less well known ^65, 66^. Enochson *et al*. demonstrated that GDF5 also represses *MMP13* expression in human articular chondrocyte pellets by inhibiting canonical Wnt signalling via DKK1 upregulation ^67^. However, Ratnayake *et al*. were unable to identify a consistent *MMP1* or *MMP13* response to GDF5 treatment in monolayer or micromass chondrocytes ^68^.

DNA methylation at *MMP13* proximal promoter CpGs has previously been shown to interfere with the binding of transcription factors CREB and HIF2α, thereby regulating gene expression ^69, 70^. RUNX2 is also able to trans-activate the *MMP13* promoter, and Takahashi *et al*. reported that the upregulation of RUNX2 in OA correlated with reduced promoter methylation, and they further confirmed a loss of methylation at numerous *MMP13* proximal promoter CpGs—in line with the upregulation of *MMP13* during OA ^71^. DNA methylation arrays assessing changes in OA compared with undamaged or healthy control tissue have identified differentially methylated CpGs at both *MMP13* and *MMP1* loci ^72, 73^, although the functional consequences of these require testing.

MicroRNAs (miRNAs) post-transcriptionally regulate gene expression, and numerous miRNAs have postulated roles in skeletal biology and regulation of chondrocyte function ^74^. *MMP13* has a relatively short 3′ untranslated region (3′-UTR) with few predicted miRNA binding sites ^75^. However, a number of miRNA interactions have been experimentally validated *in vitro*, including for miRNAs 27b-3p, 125b-5p and 140-5p ^76^. Liang *et al*. also suggested that miR-140 targets MMP-13 as a result of 17-β-estradiol (E2) stimulation increasing miR-140-5p ^77^. Liu *et al*. demonstrated that miR-136 might also target the MMP-13 3′-UTR in competition with a potential sponge circular RNA, circRNA-CER, although more evidence of these interactions is needed ^78^. In a more detailed study, Meng *et al*. reported that miR-320 is regulated by IL-1 signalling and represses *MMP13* expression directly ^79^. Consistent with previous studies, miR-320 is also upregulated during chondrogenesis which might contribute to the suppression of *MMP13* expression ^79^. Two long non-coding RNAs—lncRNA‐CIR (RP11‐162L10.1) and GAS5—have also recently been shown to regulate the expression level of *MMP13*, although again further work is necessary to establish their mechanisms of action ^80, 81^.

## Recent advances in MMP-13 protein regulation and inhibition

The MMP-13 protein is 54 kDa in size and has catalytic, linker and haemopexin domains. As described above, MMP-13 is one of only three soluble MMPs capable of triple-helical collagen cleavage with a strong preference for type II collagen ^82^, although the mechanism by which this happens is not yet fully elucidated. In recent years, crystal structures have revealed the mode of binding for the related collagenase, MMP-1 ^83, 84^, and the catalytic and haemopexin domain play essential roles in the interaction. Furthermore, novel exosites on MMP-13 which have provided insight into the mode of collagen binding have now been identified ^85^.

Regulation of MMP-13 at the protein level is multifaceted and includes activation, endocytosis and inhibition. The activation of proMMPs is of central importance in effecting cartilage degradation ^86– 89^. An exciting field of research seeks to distinguish MMP-13 activity from total protein by using specific activity-based probes. Such probes can detect MMP-13 activity in mice that have undergone DMM surgery and could provide a useful tool for detecting early changes in OA prior to significant histological damage ^90, 91^.

Identifying novel physiological activators of MMP-13 should be an important area of research, and the likely importance of serine proteinases in these processes was recently reviewed ^92^. MMP-13 can be activated directly by plasmin ^93^, and although this proteinase is not expressed by chondrocytes ^94^, diffusion from the synovium should not be discounted. *In vivo* activators of MMP-13 may include other MMPs such as MMP-3 or MMP-14 ^82, 95^. Indeed, the membrane-anchored serine proteinases matriptase and hepsin can both induce collagen release from human OA cartilage and, although neither can directly activate MMP-13, both are potent activators of MMP-3 ^94, 96^ and thus indirectly MMP-13. Jackson *et al*. demonstrated an increase in MMP-13 activity in cultures treated with activated protein C, although the authors suggested that this likely acts through an intermediary within OA cartilage matrix ^97^.

The major endogenous inhibitors for metalloproteinases are the tissue inhibitors of metalloproteases (TIMPs). Four members are expressed in human tissues (TIMP1–4), and each exhibits a two-domain structure: an N-terminal domain containing a ‘wedge-shaped’ ridge which binds to the metalloproteinase active site and a C-terminal domain which interacts with the haemopexin domain ^98^. TIMP-3 in particular has been ascribed a chondroprotective role in cartilage, and aged mice deficient in this inhibitor exhibit increased cartilage collagen destruction ^99^. TIMPs can be structurally engineered, and Lim *et al*. generated mutants of TIMP-3 which selectively inhibit a disintegrin and metalloproteinase with thrombospondin motifs 4 (ADAMTS-4) and ADAMTS-5 ^100^. This may represent a strategy to harness the natural potency of TIMPs whilst improving selectivity for particular metalloproteinases.

In recent years, our understanding of the importance of endocytosis in regulating cartilage metalloproteinase levels has grown significantly. Low-density lipoprotein-related protein-1 (LRP-1) functions as an endocytic receptor, which has been shown to have a significant effect on the levels of metalloproteinases and their inhibitors in cartilage. Indeed, ADAMTS-5, ADAMTS-4, TIMP-3 and MMP-13 are endocytosed by LRP-1 in chondrocytes ^101– 105^. Interestingly, MMP-13 bound LRP-1 via its haemopexin domain at a site distinct to ADAMTS-5, ADAMTS-4 and TIMP3, which permits co-endocytosis with these proteins ^101^. Intriguingly, proMMP-13 and active MMP-13 bound to LRP-1 with similar dissociation constants. These data suggest that selected metalloproteinases, including MMP-13, are constitutively produced by chondrocytes but their rapid endocytosis by LRP-1 may explain why they are difficult to detect in healthy adult tissue ^101^. LRP-1 shedding is increased in OA ^103^, a process which involves ADAM17 and MMP-14 ^104^, inhibition of which may represent a novel targeting strategy to reduce proteinase levels (such as those of MMP-13) in cartilage. Interestingly, a different therapeutic strategy involves reducing TIMP-3 binding to LRP-1, thereby increasing its level in the ECM. TIMP-3 mutants engineered to resist endocytosis have prolonged chondroprotective activity ^106^, and the chemical suramin, which binds to TIMP-3 and reduces its capacity to be endocytosed, has chondroprotective effects ^107^.

As mentioned, selective MMP inhibition is a challenge due to the significant overlap of active site structural architecture. In the 1990s, a flurry of activity led to the development of MMP inhibitors for the treatment of cancer metastasis, which ultimately failed because of the broad range of MMPs which they targeted ^92^. In fact, it is now recognised that a significant number of MMPs are considered ‘anti-targets’ ^19^ which must be avoided. The central importance of MMP-13 in the irreversible destruction of cartilage collagen means that it remains an attractive target for the treatment of OA, and new waves of inhibitors demonstrate remarkably improved selectivity when compared with the initial failed compounds. Indeed, many make use of the unusually large S1′ pocket in the MMP-13 active site, which is distinct amongst the MMPs. The latest advances and approaches in MMP-13 medicinal chemistry were recently reviewed elsewhere ^108^. Of course, even very specific inhibitors could have adverse effects because MMP-13 has functions outside of cartilage (for instance, during wound healing) ^13^.

Other methods outside of traditional small-molecule inhibition have also been explored. An interesting study by Naito *et al*. demonstrated the development of a novel MMP-13 neutralising antibody, which selectively binds the active MMP-13 and is highly selective over other MMPs ^109^. Antibody technology is an effective method to selectively target MMP-13 and reduce off-target effects against other MMPs. Although antibody therapeutics have disadvantages over conventional small-molecule inhibitors (such as limited administration routes, difficulties penetrating cartilage, and expense), they have proven successful for the treatment of rheumatoid arthritis and are being explored for other targets in OA ^110^. We hypothesise that there is likely to be significant research in this area with respect to MMP-13 in the coming years.

## Conclusions and future directions

In our view, MMPs, and especially the collagenases, are tractable targets for preventing cartilage destruction in OA. Although the initial wave of MMP inhibitors failed in the clinic, there does appear to be renewed interest both in terms of direct inhibition but also in targeting mechanisms of regulation as a therapeutic target, be it via activation or gene expression mechanisms. Here, we have summarised recent work examining such regulatory mechanisms and focussed on transcriptional and post-translational MMP-13 regulation.

In terms of gene regulation, we expect new discoveries on the mechanism of MMP regulation to be aided by the improvement and application of both genome-wide and single-cell technologies. Our current understanding of the role of DNA methylation in MMP-13 regulation, for example, has been limited either by the use of low-throughput technologies (such as pyrosequencing) to examine short genomic regions or by the limited presence of MMP-13 locus probes on current array-based systems. Whole genome methylation analysis will facilitate much better coverage of the MMP genomic locus, hopefully identifying important regulatory regions in disease. Similarly, our knowledge of the chromatin status of chondrocytes has largely been imputed from other cells and tissues, based on data from large-scale consortia. ChIP-seq and ATAC-seq data from chondrocytes will likely provide insight into identifying key regulatory genomic features, and systematic genomic editing (using, for example, CRISPR/Cas9) will be needed to characterise the importance of these regions. Deactivated Cas9-fusion proteins are also proving invaluable in confirming the gene regulatory function of genomic loci. As with all regulatory networks, these complex datasets will require integrated bioinformatics tools to fully delineate the mechanisms involved in MMP regulation and such a systems approach is beginning to identify underlying mechanisms involved in age-related changes in musculoskeletal tissues ^111^. Interestingly, examining correlative gene expression programmes may also lead to further understanding of gene regulatory networks; with this in mind, SkeletalVis provides an accessible data portal for comparing cross-species skeletal transcriptomics data ^112^.

Post-translational regulation of MMP-13 may also represent an attractive approach. Identifying crucial activators of MMP-13 (or indeed other MMPs upstream of MMP-13) is important, as such proteinases may represent a novel and indirect method to limit collagen degradation. Proteinases do not act in isolation but rather are the culmination of complex proteolytic cascades, of which MMP-13 is the final effector. The evolving field of ‘degradomics’ will likely prove essential for the deconvolution of such cascades and lead to the identification of novel proteinases or indeed new substrates for previously well-described proteinases. The importance of endocytosis is also becoming clear. Catabolic metalloproteinases (including MMP-13) which are not removed efficiently from the extracellular milieu by endocytic mechanisms are damaging, and efforts to ‘boost’ these mechanisms represent an interesting approach to reduce the proteolytic burden on cartilage.

MMP-13 expression and function are not limited solely to cartilage; thus, systemic delivery mechanisms to regulate MMP-13 activity, even with very specific inhibitors, may still elicit adverse effects. One intriguing possibility is delivery of anti-inflammatory or immunomodulatory compounds linked to other moieties that are released upon MMP-mediated cleavage ^113^. For example, Vessillier *et al*. used immunomodulatory peptides fused via an MMP cleavage site to the latency-associated peptide of TGF-β1 to limit inflammation in the collagen-induced arthritis model ^114^. This system elegantly uses pathologically active MMPs as the release mechanism, selectively targeting the biologically active compound to the required tissue, thus theoretically allowing systemic delivery. Interest also remains in intra-articular injection as a delivery route for disease-modifying OA treatments but this has a number of shortcomings mainly pertaining to inadequate drug delivery because of the biochemical nature of cartilage ^115^. However, improvements in intra-articular delivery have been reported with the advent of nanoparticles or nanocarriers. For example, nanoparticle-based intra-articular delivery of siRNA against NF-κB (p65 and p100) was non-immunogenic and cartilage-penetrant and reduced chondrocyte death following a non-invasive murine model of joint injury ^116^. Similarly, a single intra-articular injection an IGF1-conjugated nanocarrier reduced cell and aggrecan loss in a rat surgical model of OA ^117^. Thus, a range of options are emerging to effectively target cartilage-derived MMP-13 activity specifically. Finally, although this review has focussed specifically on cartilage and mainly on MMP-13, many of the advancements are applicable to the large repertoire of metalloproteinases and the many development and disease-related pathways in which they play a role ^118^.
